# Using the MWC model to describe heterotropic interactions in hemoglobin

**DOI:** 10.1371/journal.pone.0182871

**Published:** 2017-08-09

**Authors:** Olga Rapp, Ofer Yifrach

**Affiliations:** Department of Life Sciences and the Zlotowski Center for Neurosciences, Ben-Gurion University of the Negev, Beer Sheva, Israel; Tel Aviv University Sackler Faculty of Medicine, ISRAEL

## Abstract

Hemoglobin is a classical model allosteric protein. Research on hemoglobin parallels the development of key cooperativity and allostery concepts, such as the ‘all-or-none’ Hill formalism, the stepwise Adair binding formulation and the concerted Monod-Wymann-Changuex (MWC) allosteric model. While it is clear that the MWC model adequately describes the cooperative binding of oxygen to hemoglobin, rationalizing the effects of H^+^, CO_2_ or organophosphate ligands on hemoglobin-oxygen saturation using the same model remains controversial. According to the MWC model, allosteric ligands exert their effect on protein function by modulating the quaternary conformational transition of the protein. However, data fitting analysis of hemoglobin oxygen saturation curves in the presence or absence of inhibitory ligands persistently revealed effects on both relative oxygen affinity (*c*) and conformational changes (*L*), elementary MWC parameters. The recent realization that data fitting analysis using the traditional MWC model equation may not provide reliable estimates for *L* and *c* thus calls for a re-examination of previous data using alternative fitting strategies. In the current manuscript, we present two simple strategies for obtaining reliable estimates for MWC mechanistic parameters of hemoglobin steady-state saturation curves in cases of both evolutionary and physiological variations. Our results suggest that the simple MWC model provides a reasonable description that can also account for heterotropic interactions in hemoglobin. The results, moreover, offer a general roadmap for successful data fitting analysis using the MWC model.

## Introduction

Hemoglobin is a classical model allosteric protein, with research on this protein mirroring the development of key cooperativity and allostery concepts [1–7]. The steady-state sigmoidal profile of oxygen binding to hemoglobin provided the basis for the ‘all-or-none’ Hill formulation offered in 1910 [8]. Fifteen years later, Adair proposed a phenomenological stepwise binding mechanism to account for hemoglobin saturation [9]. A seminal paper by Linus Pauling published ten years later was the first to suggest a structural or geometry-oriented explanation for cooperative oxygen binding by hemoglobin. Pauling constructed a grand partition function to fit Adair's data using a simple sequential model with a single oxygen binding constant and a single heme-heme interaction parameter [10]. Later attempts to rationalize the cooperative binding of oxygen to hemoglobin relied on the Monod-Wymann-Changuèx (MWC) [11–12] and the Pauling-inspired Koshland-Nemethy-Filmer (KNF) [13] mechanistic models, both developed in the mid-1960’s and respectively involving concerted and sequential subunit transitions. It subsequently became apparent that the MWC model (S1 Fig (panel A)) better describes hemoglobin function. In particular, hemoglobin was found to exist in equilibrium between the deoxy and oxy conformations, corresponding to the structural correlates of the respective **T** and **R** quaternary conformations of the MWC formulation [14–16]. Second, either conformation, when isolated in the crystal [17–18] or gel phase [19–20], binds four oxygen molecules in an independent (hyperbolic) manner, albeit with distinct affinities (*K*_T_ and *K*_R_, respectively). Other evidence, summarized in numerous reviews (*e*.*g*. [6, 21]), indicated that the MWC model adequately accounts for homotropic interactions in hemoglobin involving the four distant O_2_-binding sites.

A different picture, however, emerges when attempting to apply the MWC model in explaining heterotropic interactions of hemoglobin. According to the MWC model, allosteric ligands, whether activating or inhibitory, only affect the **T** to **R** quaternary conformational equilibrium of the protein (*L* = [**T**]/[**R**]) [11–12]. Indeed, Edelstein (1971) pointed out that the alkaline Bohr effect of hemoglobin with its associated ‘buffering of cooperativity’ phenomena (i.e., the observation that pH changes affect primarily oxygen affinity (*p*_50_) with no change in cooperativity (*n*_H_); see S1 Fig (panel B)) can be explained by proton-induced changes in *L* [22]. However, attempts to fit steady-state saturation curves of hemoglobin in the presence of its H^+^, CO_2_ or organophosphate inhibitors to the MWC equation, assuming *L*-only effects, consistently failed. Rather, successful fits to such physiological data were obtained only when both *L* and the *c* (= *K*_R_/*K*_T_) parameters were allowed to change [23–27]. These steady-state observations called for modifications of the original MWC model to include additional quaternary conformational states for hemoglobin [24, 28] or tertiary interactions [29–31]. Hemoglobin was no longer considered a ‘pure’ MWC protein (see Discussion). Still, several points regarding the curve fitting procedure remained problematic. First, large error bars were usually obtained for evaluated parameters, especially for *L* [23], indicating that the data, even if accurate and intensively sampled, did not constrain parameter values and that other parameter sets could also yield a successful fit. Second, the values obtained for the *L* and *c* parameters of a concentration-related physiological dataset often correlated without an intuitive mechanistic explanation [29,31–32]. Finally, in many cases, the derived *L* or *c* values failed to scale with effector concentration, instead appearing to be artificially correlated (see, for example, the rigorous physiological datasets presented and analyzed in S2 Fig, addressing the Bohr effect of hemoglobin in the presence of different organophosphate inhibitors [29]). These observations prompted suggestions that hemoglobin saturation data can be described by only two [31], and later, by even one [32] of the MWC parameters. It can be argued that these points contributed to the common notion that estimates for MWC parameters, in particular *L*, are not always reliable and should be viewed with care.

The elegant meta-analysis study of hemoglobin function by Milo *et al*. [33] addressed this issue in a rigorous manner. The study revealed that due to the different sensitivities of the *L*, *K*_R_ and *K*_T_ parameters to experimental variation, values for these parameters could not be unequivocally determined using the traditional MWC equation (see Fig 3 in reference [33]). To overcome this shortcoming, the authors presented a modified form of the MWC equation, re-parameterized based on the *Lc*^4^ and *LK*_R_^4^ compound parameters. Fitting steady-state oxygen-binding data to the modified equation yielded reliable estimates for the *LK*_R_^4^ and *Lc*^4^ hemoglobin parameters [33]. Considering that these parameters are respectively related to the midpoint transition point (*P*_50_) and cooperativity (*n*_H_) phenotypic parameters of the binding curve [33], use of the modified version of the MWC equation makes intuitive sense. Still, the modified equation cannot provide estimates for the elementary *L*, *K*_R_ and *K*_T_ MWC parameters of hemoglobin.

The *L*, *K*_R_ and *K*_T_ parameters are related to concrete, easy-to-interpret steps in the MWC ligation pathway [11]. Moreover, knowing the values of these parameters is essential for understanding how physiological changes, mutations and evolutionary variations affect hemoglobin function. In this manuscript, we present two simple strategies for obtaining reliable estimates of *L*, *K*_R_ and *K*_T_ for both physiological and evolutionary variations and offer criteria for assessing the performance of each strategy. Using the extensive physiological and evolutionary functional datasets available for the hemoglobin model allosteric protein previously compiled [33], we demonstrate the usefulness of the strategies described here for obtaining reliable mechanistic knowledge on hemoglobin. Our results suggest that both homotropic and heterotropic interactions are adequately described by the traditional MWC model. Furthermore, the present study delineates a general roadmap for the successful fitting of steady-state ligand binding data to the MWC allosteric model to yield reliable estimates for the mechanistic parameters of the protein of interest.

## Materials and methods

### Datasets

The functional data analyzed here considered hemoglobin physiological and evolutionary datasets [32–33, 34–36]. The evolutionary dataset comprised oxygen saturation curves of hemoglobin from 27 different mammal samples obtained under similar physiological conditions and at room temperatures (~25°C) [33]. The physiological dataset comprised four independent human hemoglobin sub-datasets, obtained from three different labs, two reporting pH-based modulation [32,34], one considering CO_2_-based modulation [35] and the last addressing 2,3-BPG-based modulation [36]. These sub-datasets each include between 5–7 oxygen saturation curves obtained at different concentrations of the same effector and were collected at a temperature range of 20–30°C, as indicated in the appropriate reference.

### Solving a three-unknown equation system

To extract reliable estimates for the *K*_T_, *K*_R_ and *L* mechanistic MWC parameters of all hemoglobin saturation curves in both the physiological and evolutionary datasets, the following three-unknown equation system was used: y_1_ (*L*, *K*_R_) = *LK*_R_^4^; y_2_ (*L*, *K*_R_, *K*_T_) = *L(K*_R_/ *K*_T_)^4^; ${\text{y}}_{3}={\bar{Y}}_{MWC}^{half\;saturation}\;(L,K_{\text{R}},K_{\text{T}},P_{50})$. Using the list of y_1_, y_2_ and *P*_50_ values reported for each hemoglobin saturation curve in the datasets [33], this quadratic equation system is analytically solvable to yield two real and two non-real roots. As elaborated in the main text, only one of the real roots solution sets is physiologically sound. Errors in *K*_T_, *K*_R_ and *L* were calculated using standard error propagation based on the reported errors in *LK*_R_^4^, *Lc*^4^ and *P*_50_ (when available) and according to the following general equation for error propagation (Eq 1):

$$\Delta f(x,y,\ldots )=\sqrt{\frac{\delta f(x,y,\ldots )}{\delta x}\Delta x+\frac{\delta f(x,y,\ldots )}{\delta y}\Delta y+\ldots }$$ (1)

### Data fitting

Fractional saturation ($\bar{Y}$) data were fitted using either the Hill or MWC equations (Eqs 2 and 3 below, respectively) and the adequacy of fit was judged based on attaining a *R*^2^ correlation coefficient greater than 0.97.

$$Y=\frac{P_{O_{2}}^{n_{H}}}{P_{O_{2}}^{n_{H}}+P_{50}^{n_{H}}}$$ (2)

$$Y_{MWC}=\frac{\frac{P_{O_{2}}}{K_{R}}{\left(1+\frac{P_{O_{2}}}{K_{R}}\right)}^{3}+L\frac{P_{O_{2}}}{K_{T}}{\left(1+\frac{P_{O_{2}}}{K_{T}}\right)}^{3}}{{\left(1+\frac{P_{O_{2}}}{K_{R}}\right)}^{4}+L{\left(1+\frac{P_{O_{2}}}{K_{T}}\right)}^{4}}$$ (3)

In the case of MWC analysis, global fitting analysis of all hemoglobin oxygen saturation curves in the physiological datasets (obtained at different effector concentrations) was performed (OriginPro 2015 software, OriginLab). In such analysis, all effector saturation curves are simultaneously fitted to the classical form of the MWC equation to obtain a single estimate for the *K*_R_ and *K*_T_ value pair and varying *L* values for the different curves.

The dependence of *n*_*H*_ at half-saturation ($n_{H}^{MWC}$) on MWC mechanistic parameters (See S1 Fig) was obtained by applying the Hill transformation to the MWC equation (using Matlab package software), assuming ${\bar{Y}}_{MWC}$ equals ½, as described by Eq 4 and in ref. [37].

$$n_{H}^{MWC}=\frac{\partial ({\bar{Y}}_{MWC}/1-{\bar{Y}}_{MWC})}{\partial (\text{log}[S])}$$ (4)

## Results

### The three-equation system strategy

How can reliable estimates for the *L*, *K*_R_ and *K*_T_ MWC mechanistic parameters of hemoglobin saturation curves be obtained? As pointed out above, reliable estimates for the *Lc*^*4*^ and *LK*_R_^4^ compound parameters can be obtained by fitting hermoglobin saturation data ($\bar{Y}$ as a function of [S]) to a modified MWC equation [33]. The *Lc*^4^ and *LK*_R_^4^ parameters, with their estimated values, represent two equations with three unknowns. Realizing the values of the three *L*, *K*_R_ and *K*_T_ parameters of each hemoglobin saturation curve in the datasets thus requires a third equation. Using the initial condition of half-saturation (where ${\bar{Y}}_{MWC}\;=\;½$), we added a third expression delineating the dependence of the MWC transition midpoint ([*P*_*50*_]^MWC^) on the *L*, *K*_R_ and *K*_T_ model parameters. A three-equation system (TES) with three unknowns is thus obtained that is tractable for an analytical solution (see Methods). The extensive evolutionary and physiological datasets for hemoglobin compiled in the meta-analysis of hemoglobin function [33] offer the possibility for testing the performance of the TES strategy. Estimates for the values of the compound *Lc*^4^ and *LK*_R_^4^ and *p*_*50*_ parameters for 27 mammalian hemoglobin oxygen saturation curves, all obtained under similar physiological conditions (the evolutionary dataset), and for oxygen saturation curves of human hemoglobin obtained in a variety of experimental conditions (*i*.*e*., different pH, CO_2_ pressure and 2,3-bisphosphoglycerate (2,3-BPG) concentrations; the physiological dataset) were previously reported [33].

### The TES strategy yields reliable estimates for the MWC parameters of hemoglobin evolutionary dataset curves

The above strategy was initially applied to the hemoglobin evolutionary dataset. Each mammalian hemoglobin oxygen saturation curve is characterized by the three equations above, with their y_1_, y_2_ and y_3_ values listed in ref. [33]. Solving such a three-equation system for each mammalian hemoglobin in the dataset should yield up to four roots for each parameter, as expected for an equation system to the fourth power. Indeed, of the 27 equation systems solved for the evolutionary dataset, 17 presented two distinctive real roots and two non-real roots. An additional 10 systems presented only one real root and two imaginary roots, apparently reflecting convergence of the two real roots. Values for the two real roots of the *L*, *K*_R_ and *K*_T_ parameters for all 27 dataset saturation curves are reported in Table 1. As can be seen, the two real solution sets are clearly distinctive of each other. While one set revealed *L*, *K*_R_ and *K*_T_ values that seem physiologically sound (Table 1, right columns in red), the other did not (Table 1, left columns). For example, the hemoglobin *L* values of the physiologically sound solution set ranged from 10^4^−10^9^, as typically reported in the literature [3,11], whereas those of the non-physiological set all clustered around *L* = ~1 (Table 1). The same is true for the relative affinity *c* parameter (= *K*_R_/*K*_T_). Whereas in the physiologically sound set, the obtained range of *c* values (10^−2^–10^−3^) was compatible with reported values for hemoglobin [3,11], the non-physiological solution set exhibited much higher affinity ratio values for almost all curves, in the range of 0.1–0.3 (Table 1). Furthermore, the 17 mammalian systems comprising the physiologically-sound solution set contained three independent triplicates for human hemoglobin, obtained from different labs (see references within Table 1), thus providing an internal control to validate our strategy. As can be seen in Table 1, relatively similar *L* and *c* values were obtained for each repeat in the human triplicates that are in good agreement with those reported in the literature (~10^6^ and ~0.01) [3–4,22].

**Table 1:** ‘TES strategy’-derived MWC parameters of mammal hemoglobin oxygen saturation curves of the evolutionary dataset^a^.

			Non-physiological solution set				Physiologically-sound solution set				
#	^b^Mammalian Species	^c^*K*_T_ (mmHg)	*K*_R_ (mmHg)	*c*	*L*	*n*_H_ ^d^Calculated (MWC)	*K*_R_ (mmHg)	*c*	*L* x(10^5^)	*n*_H_ ^d^Calculated (MWC)	*n*_H_ ^e^Observed
1	*African elephant 1*	102.3±2.7	18.2±0.1	0.178±0.005	1.6±0.2	1.18±0.01	0.5±0.1	0.005 ±0.001	30.2±3.2	2.85±0.20	2.84±0.07
2	*African elephant 2*	138.0±12.4	18.3±0.5	0.133±0.012	2.0±0.7	1.23±0.06	1.6±0.2	0.011 ±0.002	0.4±0.1	2.87±0.17	2.92±0.17
3	*Asian elephant*	63.1±2.4	19.4±0.1	0.308±0.012	1.1±0.2	1.18±0.05	0.3±0.2	0.003 ±0.001	240.3±92.5	2.88±0.80	3.01±0.16
4	*Horse*	112.2±1.3	19.3±0.1	0.172±0.002	1.8±0.1	1.11±0.01	1.4±0.5	0.012 ±0.006	0.7±0.6	2.74±0.58	2.76±0.02
5	*Camel*	72.4±1.1	19.3±0.2	0.266±0.005	2.0±0.1	1.20±0.01	2.6±0.1	0.036 ±0.001	0.1±0.1	2.20±0.02	2.26±0.05
6	*Cow*	89.1±0.6	20.6±0.1	0.232±0.002	1.4±0.0	1.19±0.01	0.2±0.1	0.002 ±0.001	1565.4±9.7	2.67±0.27	2.87±0.05
7	*Mole*	91.2±2.0	20.7±0.1	0.226±0.005	1.5±0.1	1.18±0.01	0.7±0.1	0.008 ±0.001	9.5±0.3	2.61±0.06	2.68±0.1
8	*Orangutan*	120.2±8.4	21.4±0.3	0.178±0.013	1.6±0.4	1.15±0.01	0.7±0.2	0.006 ±0.002	16.6±6.2	2.84±0.48	2.76±0.09
9	*Gorilla-F*	93.3±2.1	21.7±0.2	0.232±0.006	1.4±0.1	1.18±0.01	0.1±0.1	0.002 ±0.001	7457.4±86.7	2.68±0.78	2.72±0.16
10	*Chimpanzee*	95.5±2.0	21.7±0.2	0.227±0.005	1.5±0.1	1.16±0.01	0.7±0.1	0.008 ±0.001	12±1.1	2.62±0.20	2.87±0.08
11	*Platypus*	158.5±1.6	21.5±0.1	0.135±0.002	1.9±0.1	1.18±0.04	1.3±0.3	0.008 ±0.003	1.3±0.9	2.94±0.33	3.18±0.04
12	*Human* (Clerbaux *et al*)^f^	123.0±1.3	22.0±0.1	0.179±0.002	1.5±0.1	1.15±0.01	0.5±0.1	0.004 ±0.001	62.1±13.9	2.87±0.40	2.77±0.06
13	*Human* (Severinghaus *et al*)^f^	125.9±0.6	22.0±0.0	0.175±0.001	1.7±0.0	1.16±0.01	1.1±0.1	0.009 ±0.001	2.5±0.5	2.79±0.18	2.93±0.03
14	*Human* (Imai) ^f^	128.8±0.8	22.1±0.1	0.171±0.001	1.8±0.0	1.22±0.01	1.6±0.1	0.013 ±0.001	0.7±0.1	2.74±0.04	2.91±0.02
15	*Antelope*	338.8±37.0	23.0±0.3	0.068±0.007	1.9±0.8	1.18±0.01	0.5±0.5	0.002 ±0.002	83.4±142.8	3.46±0.80	3.59±0.15
16	*Goat*	436.5±26.9	23.5±0.1	0.054±0.003	1.9±0.5	1.19±0.01	0.4±0.5	0.001 ±0.001	274.5±494.2	3.57±0.78	3.54±0.11
17	*Dog*	104.7±1.0	23.7±0.1	0.226±0.002	1.5±0.1	1.20±0.01	0.8±0.1	0.008 ±0.001	9.2±0.1	2.61±0.07	2.62±0.06
18	*Llama*	97.7±0.7	23.0±0.1	0.235±0.002	1.3±0.0	1.14±0.01	NA	NA	NA	NA	2.90±0.06
19	*Yak*	338.8±37.0	23.0±0.3	0.068±0.007	1.9±0.8	1.23±0.08	NA	NA	NA	NA	3.59±0.15
20	*Gorilla-M*	144.5±1.9	21.0±0.1	0.145±0.002	1.4±0.1	1.24±0.05	NA	NA	NA	NA	3.54±0.11
21	*Echidna*	97.7±0.7	23.0±0.1	0.235±0.002	1.3±0.0	1.16±0.01	NA	NA	NA	NA	2.62±0.06
22	*Deer*	131.8±2.2	24.1±0.1	0.183±0.003	1.4±0.1	1.17±0.01	NA	NA	NA	NA	2.99±0.07
23	*Shrew*	239.9±2.4	27.7±0.0	0.115±0.001	1.4±0.1	1.18±0.01	NA	NA	NA	NA	3.30±0.06
24	*Hedgehog*	208.9±1.8	30.6±0.0	0.146±0.001	1.4±0.0	1.17±0.01	NA	NA	NA	NA	3.15±0.04
25	*Ringtailed lemur*	213.8±13.7	30.9±0.2	0.144±0.009	1.4±0.4	1.18±0.04	NA	NA	NA	NA	3.29±0.19
26	*Brown galago*	131.8±5.5	30.8±0.3	0.234±0.001	1.3±0.2	1.15±0.02	NA	NA	NA	NA	2.91±0.14
27	*Black galago*	166.0±9.3	31.4±0.2	0.189±0.011	1.2±0.3	1.15±0.03	NA	NA	NA	NA	2.94±0.14

^*a*^The MWC allosteric parameters reported in this table for hemoglobin evolutionary dataset were obtained using the TES strategy, as described in the main text. The *K*_T_ and *K*_R_ parameters correspond to the affinity of oxygen to the respective **T** and **R** hemoglobin MWC quaternary states, whereas *c* and *L* correspond to the relative affinity (c = *K*_R_/ *K*_T_) and conformational stability (*L* = [**T**]/[**R**]) of the **T** and **R** states.

^*b*^The mammalian hemoglobin species analyzed in the current meta-analysis, as reported in ref. [33].

^c^ For each hemoglobin species, the value for *K*_T_ is unequivocally determined (using the *LK*_R_^4^ and *Lc*^4^ values reported in ref. [33]) and is valid for both physiological and non-physiological solution sets.

^*d*^Hill coefficient at half-saturation calculated based on the *K*_R_, *K*_T_, and *L* MWC parameters (see Methods and reference [37]).

^*e*^Hill coefficients at half-saturation obtained upon fitting hemoglobin oxygen saturation data to the Hill equation (Eq 2), as reported in ref. [33].

^*f*^Source data for the different human proteins is reported in the general reference list of ref. [33].

**Fig 1:**
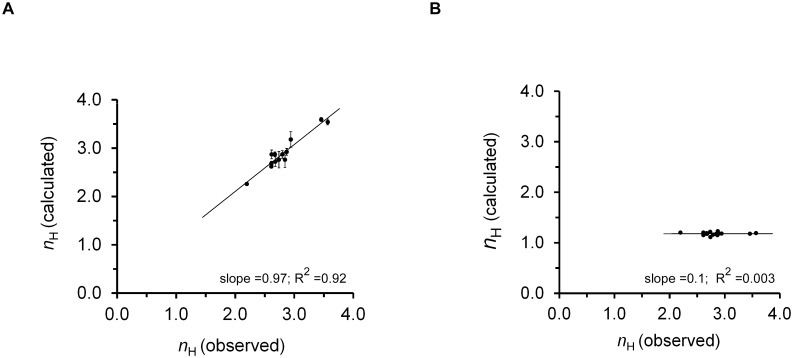
Successful application of the three-equation system (TES) strategy in the case of evolutionary variations in hemoglobin. (A) Correlation plot relating the values of *n*_H_ for the 13 different mammalian hemoglobins of the physiologically-sound solution set derived either using the Hill equation (as reported in ref. [33]) or calculated according to the MWC model, based on *L*, *K*_R_ and *K*_T_ values (Table 1, red columns). Values for the human and elephant species correspond to the averaged values of three independent triplicates. (B) The same analysis but for the non-physiological solution set (Table 1, rightmost black columns). The expression for *n*_H_ in terms of the *L*, *K*_R_ and *K*_T_ model parameters is known and can be obtained using the $\partial ({\bar{Y}}_{MWC}/1-{\bar{Y}}_{MWC})/\partial \left(\text{log}\left[S\right]\right)$ Hill transformation (see Eq 4 and Methods).

Despite the above arguments implying the relevance of the TES strategy employed, we sought an independent measure that would confirm the validity of our strategy and allow us to further distinguish between physiologically and non-physiologically sound solution sets. Such a measure is obtained upon comparison of the observed Hill coefficient values of different mammalian hemoglobins (*n*_H_), as reported [33], to those calculated according to the MWC model (*n*_H_^MWC^) [37], based on the derived *L*, *K*_R_ and *K*_T_ values of either set (Fig 1; see Eq 4 in Methods). Whereas linear correlation with a slope close to unity (0.97) was observed for the physiologically-relevant solution set (Fig 1A; *R*^2^ = 0.92), no correlation at all was noted for the non-physiological solution set (Fig 1B; *R*^2^ = 0.337). All non-physiological parameter sets gave rise to non-physiological Hill values, all close to 1. These findings indicated that our mathematical strategy for obtaining *L*, *K*_R_ and *K*_T_ values for the evolutionary dataset saturation curves, based on *Lc*^4^, *LK*_R_^4^ data and the *p*_50_ constraint, is indeed valid. This further enabled us to distinguish between physiological and non-physiological mathematical solutions for the microscopic parameters, stemming from the dimensionality of the problem.

Next, we considered whether the TES strategy is also applicable to the physiological dataset. Consider, for example, the Bohr effect of hemoglobin [4,38]. Can the estimates of *L*, *K*_R_ and *K*_T_ obtained by employing the TES strategy with all dataset curves rationalize the Bohr effect of hemoglobin? Traditionally, in the context of the MWC model [11], and as further emphasized in detail by Rubin and Changeux [12], physiological effects on protein function are brought about by changes in the quaternary conformational equilibrium (*L*-only effects). Furthermore, the explicit theoretical dependence of the apparent *L* constant on effector concentration is known [11,12]. Still, the modified MWC equation based on compound *Lc*^4^ and *LK*_R_^4^ parameters [33] cannot be *a priori* bound to *L*-only effects. Moreover, for any effector dataset, each *L* and *c* value pair is obtained by solving an equation system for each oxygen saturation curve of the physiological dataset separately, ignoring the relation between the oxygenation curves obtained at different concentrations of the same effector. Yet, even with these concerns, a solid criterion to assess the performance of the TES strategy is available; either of the derived *L* or *c* parameters (or both) should scale with effector concentration. No matter the ligation scheme considered and the manner by which the effect of modulatory allosteric ligands is implemented, the apparent effect of the ligands on any affected scheme parameter should be concentration-dependent and monotonic in nature. This is a mechanistic coherence argument that makes intuitive sense.

**Fig 2:**
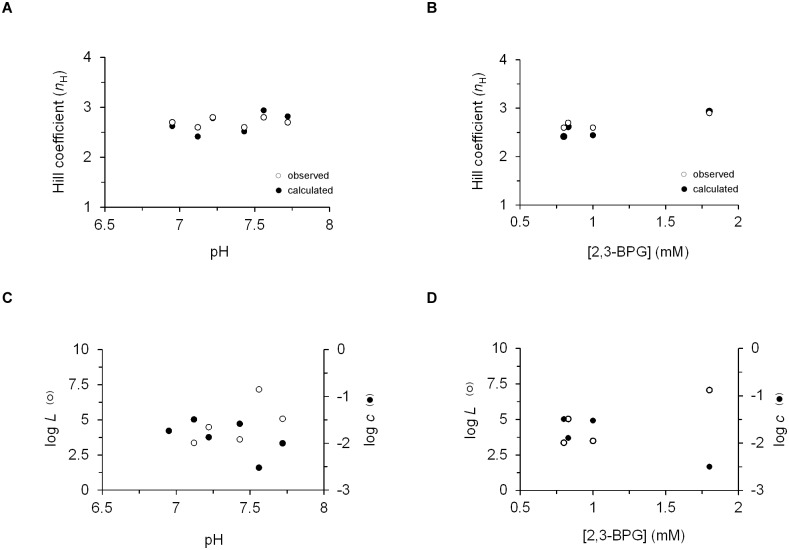
The three-equation system strategy is inadequate for assessing the effect(s) of physiological variations on allosteric protein function. (A-B) Correlation plot relating the observed (open circles) and calculated (filled circles) *n*_H_ values of the different dataset oxygenation curves to either pH (A) or 2,3-BPG (B) effector concentrations (S1 Table; see Methods). (C-D) Dependence of the *L* and *c* parameters of the physiological datasets on pH (C) and 2,3-BPG concentrations (D). A similar analysis of additional pH and 2,3-BPG physiological datasets, collected under different experimental conditions (see S1 Table), is presented in S2 Fig.

With this in mind, we employed the reported values for the *Lc*^4^, *LK*_R_^4^ and *p*_*50*_ parameters for all human hemoglobin oxygen saturation curves in four complete pH or [2,3-BPG] concentration-dependent physiological datasets [33] to calculate values for *L* and *c* for each curve. The results are summarized in S1 Table and in Fig 2 and S3 Fig, each describing a pair of complete pH and [2,3-BPG] datasets. Several points should, however, be noted. First, here again, as in the case of the evolutionary dataset, physiologically-sound and non-sound parameter sets were obtained (not shown). As above, we only relate to the physiologically sound set (S1 Table). Second, for all pH or 2,3-BPG physiological datasets, unlike the expectations of the MWC model [11,12], changes are observed in both *L* and *c* (S1 Table). Furthermore, for all four datasets, the derived *L* and *c* values give rise to Hill values that are relatively similar to those obtained using the Hill equation (Fig 2A and 2B and S3 Fig (panels AB)), seemingly reflecting the reliability of the estimated parameters. However, when plotting for each effector dataset, the dependence of either the apparent *L* constant (open circles) or the relative affinity *c* constant (filled circles) on effector concentration ([H^+^] or [2,3-BPG]), no monotonic trend between either of the two quantity pairs was obtained (Fig 2C and 2D and S3 Fig (panels CD)). In other words, the *L* and *c* parameters obtained using the TES strategy do not scale with effector concentration. In fact, it seems that both estimated parameters are reciprocally adjusted. Combined, our results confirm our concerns that, unlike in the case of the evolutionary dataset, the three-equation system strategy, although mathematically valid, cannot be employed for physiological datasets involving *L*-only effects, as commonly treated by the MWC model [11,12]. In what follows, we address how reliable estimates for these values can be obtained.

### Global fitting analysis of hemoglobin physiological datasets yields reliable estimates for MWC parameters

**Fig 3:**
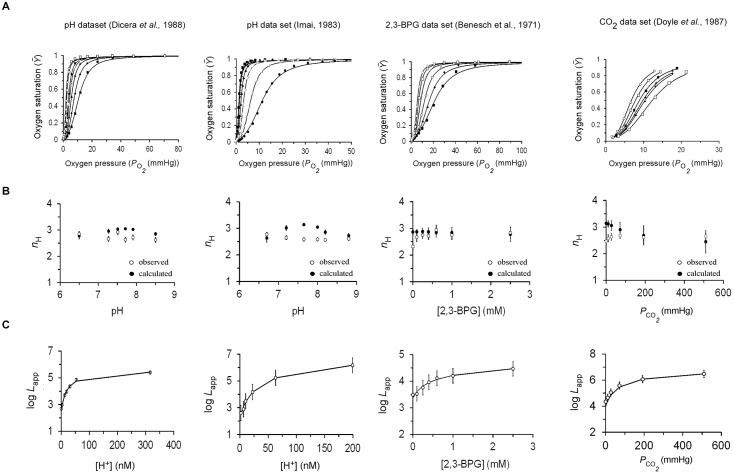
Successful application of global fitting analysis of hemoglobin physiological datasets. (A) Global fitting analysis of the human hemoglobin pH, CO_2_ and 2,3-BPG physiological datasets using the traditional form of the MWC equation. Source data is indicated above each panel. (B) Dependence of observed (open circles) and calculated (filled circles) Hill values of each dataset on effector concentration. (C) Dependence of the apparent *L* values of each physiological dataset on effector concentration. Solid curves represent the results of curve fitting to the MWC-derived equation $L_{app}=L_{o}{\left((1+\left[I\right]/K_{I}^{T})/(1+\left[I\right]/K_{I}^{R})\right)}^{4}$, assuming non-exclusive binding of the inhibitor effector (I) to both the **T** and **R** MWC conformations [11,12]. In the case of organophosphate inhibitors, a power of one was used in the above equation, as only one site is available to BPG for binding to hemoglobin.

A possible method for obtaining reliable estimates of the *L*, *K*_R_ and *K*_T_ parameters of concentration-related ligand-binding saturation curves is global fitting. In such analysis, all saturation curves, obtained at different concentrations of the same effector, are simultaneously fitted to the classical form of the MWC equation to obtain a single estimate for the *K*_R_ and *K*_T_ value pair and varying *L* values for the different curves. Such a scenario adheres to how physiological effects are treated within the MWC model [11,12], while overcoming the shortages of the classical MWC equation when employed to fit a single binding curve [33]. Surprisingly, such analysis has not been regularly employed for hemoglobin. We thus employed global fitting analysis of the solid body of hemoglobin physiological data obtained at varying concentrations of H^+^, CO_2_ and 2,3-BPG. Four physiological datasets were analyzed: Two pH datasets of Imai [32] and Di Cera *et al* [34], respectively comprising six and seven oxygen saturation curves obtained at varying pH values, a CO_2_ physiological dataset also from the Gill lab comprising six oxygen saturation curves [35] and a 2,3-BPG dataset comprising six curves [36]. The results of such global fitting analysis of each dataset performed separately are summarized in Fig 3 and S2 Table. As can be seen, all physiological datasets were successfully fitted to the MWC equation (Fig 3A), yielding a single estimate for *c* (= *K*_R_/*K*_T_) and varying apparent *L* values for the different concentration-related curves in the dataset (S2 Table). It is worth noting that the datasets, obtained by three different labs and using different hemoglobin effectors, yielded relatively similar *c* values (S3 Table) that matched those obtained for human hemoglobin using the evolutionary dataset and the TES strategy described above (Table 1). These values are similar to those reported in the literature [3–4,22]. Furthermore, for all datasets, both observed (Hill space) and calculated (MWC space) *n*_H_ values showed similar dependence on effector concentration (Fig 3B). This finding provides further support for the reliability of the global fitting analysis employed here for the physiological datasets. Finally, when the dependence of the apparent *L* constant (*L*_app_) of each oxygen saturation curve is plotted as a function of effector concentration, a monotonic dependence is observed for each case (Fig 3C). The higher the H^+^, CO_2_ or 2,3-BPG concentration, the greater are the values for *L*_app_, as expected for allosteric inhibitors (I) (Fig 3C and S2 Table). Furthermore, for each effector dataset, the data is very well fitted (*R*^2^ = 0.99) to the theoretical, MWC-derived equation that delineates the dependence of *L*_app_ on effector concentration, assuming non-exclusive ligand binding (see Fig 3 legend and ref. [11–12]), yielding estimates for *L*_*0*_ (at zero effector concentration) and the effector affinity for the **T** and **R** hemoglobin conformations ($K_{I}^{T}$ and $K_{I}^{R}$, respectively), as listed in Table 2. It should be noted that although Monod *et al*. did not treat H^+^ as a protein ligand [11] and the fact that several residues contribute to the observed Bohr effect [4,14–16], we nonetheless used the MWC framework to obtain an approximation of apparent binding constants of protons to hemoglobin conformations. As can be seen in Table 2, similar *L*_*0*_ and ($d\;\left(=K_{I}^{R}/K_{I}^{T}\right)$) values were obtained for both independent pH datasets. The low *L*_*0*_ value obtained for hemoglobin at [H^+^] = 0 and the finding that protons bind more than an order of magnitude better to the **T** conformation are also of note.

**Table 2:** ‘Global fitting’-derived MWC parameter sets for human hemoglobin pH, CO_2_ and 2,3-BPG physiological datasets^a^.

Physiology dataset	*L*_0_	*K*_I_^T^	*K*_I_^R^	*d = K*_I_^R^*/K*_I_^T^	^b^Reference
**pH**	564.5±84.8	15.1±1.3 (nM)	146.8±17.1 (nM)	9.7±1.4	Di Cera *et al*. (1988)
**pH**	230.3±13.5	9.6±0.2 (nM)	140.5±0.9 (nM)	14.6±0.3	Imai (1983)
**2,3-BPG**	2342.7±600.1	0.12±0.04 (mM)	3.8±1.1 (mM)	31.7±14	Benesch *et al*. (1971)
**CO**_**2**_	39940.8±9005.3	54.5±6.6 (Torr)	203.0±16.0 (Torr)	3.7±0.5	Doyle *et al*. (1987)

^*a*^The MWC allosteric parameters for the hemoglobin pH, CO_2_ and 2,3-BPG datasets were derived assuming a non-exclusive ligand binding mode, as described by the equation reported in Fig 3 legend and in reference [12]. *L*_0_ corresponds to the [**T**]/[**R**] ratio in the absence of allosteric effector (S1 Fig). *K*_I_^T^ and *K*_I_^R^ respectively correspond to the allosteric inhibitor affinity towards the **T** and **R** hemoglobin states and *d* to the ratio between the two affinities.

^*b*^The full reference is indicated in S2 Table, right-most column.

In addition, the analysis using the 2,3-BPG and CO_2_ datasets revealed hemoglobin *L*_*0*_ values that are coherent with reported values (given the working pH; Table 2) [3–4,22] and with the values obtained using the evolutionary dataset. Moreover, the *d* value reported for 2,3-BPG (~30) is very similar to the reported value (~20), evaluated directly without using the MWC framework [36]. Lastly, the *d* value reported here for CO_2_ (~4) is almost identical with the reported value in the literature (4.5, as reported in ref. [35]).

Combined, our studies of the hemoglobin model allosteric protein indicate that global fitting analysis is a powerful method for obtaining reliable estimates for the *c* and *L* values of physiological dataset-related substrate-binding curves obtained in the presence of different concentrations of effector ligand. The global fitting strategy further allows assessment of the affinity of allosteric inhibitors or activators to both MWC **T** and **R** quaternary states.

## Discussion

The 2007 meta-analysis of hemoglobin function by Milo *et al*. [33] highlighted the limitations of the traditional MWC equation when used to fit hemoglobin saturation data and called for a re-examination of previous data. Although providing a partial remedy to the problem, that analysis was not able to provide estimates for the *L*, *K*_R_ and *K*_T_ parameters of the different hemoglobin saturation curves. By employing the simple TES and global fitting strategies, we showed here how to obtain reliable estimates for the MWC parameter set for both hemoglobin steady-state evolutionary and physiological datasets. Our results (Figs 1–3) indicate that steady-state oxygen saturation measurements of solution hemoglobin in the presence or absence of allosteric effectors can be adequately described by the simple MWC framework, assuming that allosteric effectors exert their function by affecting only the **T** to **R** quaternary conformational equilibrium [11,12]. It thus seems that the MWC allosteric model adequately describes both homotropic and heterotropic interactions in hemoglobin.

This conclusion is based on steady-state effects of ligand binding to hemoglobin. Transient kinetics analysis imposes more restrictions on potential mechanisms that could fully account for the data obtained. The recent nanosecond-scale kinetic analysis of carbon monoxide (CO)-binding to silica gel-entrapped **T** or **R** hemoglobin conformations [39–40] has revised our understanding of the allosteric regulation of hemoglobin function. Using a laser photolysis setup with hemoglobin, these authors demonstrated that hemoglobin subunits in the **T** quaternary state can bind CO at the same fast rate as do subunits in the **R** quaternary structure. Likewise, subunits of the **R** state bind CO with the characteristic slow rate of subunits in the **T** state. Based on these results, the Eaton group suggested a natural extension to the MWC model to also include pre-equilibrium tertiary interactions [39–40]. This model, termed the tertiary two-state model (TTS), considers hybrid hemoglobin species with mixed *t* and *r* subunit conformations, and was found to be superior in explaining hemoglobin functional data over other models [41–42], including the classical two-state MWC model. Still, even with this more accurate description that accounts for hemoglobin kinetics, our data suggest that the traditional MWC model provides a reasonable first-order approximation to explain steady-state physiological effects on hemoglobin saturation. Such approximation can provide initial estimates of the ‘quaternary contribution’ (*L’*) of allosteric effectors within the framework of the TTS model, not previously possible, due to data fitting difficulties. The strategies reported here, when combined with detailed structural knowledge, can be used to rationalize molecular adaptation effects of hemoglobin in response to the ecological niches occupied by differences species, as previously reported [43–46].

The results described here offer a general data-fitting scheme for obtaining reliable estimates for the MWC mechanistic parameters of an allosteric protein. We suggest that for cases whereby variations in MWC allosteric protein functions are expected to affect both the relative affinity (*c*) and conformational equilibrium (*L*) allosteric parameters, as in the case of mammal hemoglobin evolutionary dataset curves or upon mutation, the modified form of the MWC equation should be used [33] (Fig 1). This modified form of the MWC equation, based on the *Lc*^n^ and *LK*_R_^n^ compound parameters, is suitable for constraining the data when fitted to a single binding curve [33]. Reliable estimates for *K*_R_, *K*_T_ and *L* can then be obtained by adding the initial condition of half-saturation and solving a three-equations system with three unknowns, as described in detail above. On the other hand, for cases where variations are expected to affect only *L*, as in the case of the extensive physiological datasets obtained at different concentrations of the same effector, the modified equation should not be used, as it cannot *a priori* be linked to *L*-only effects (Fig 2). Instead, a global fitting analysis based on the classical form of the MWC equation should be used (Fig 3). Such analysis succeeds in obtaining reliable estimates for *K*_R_, *K*_T_ and *L* that effectively constrain the multiple curves binding data and overcomes shortcomings associated with the traditional form of the MWC equation when employed to fit a single binding curve. This strategy further provides the affinities of allosteric inhibitors and activators for the **T** and **R** protein conformations (Table 2).

To summarize, the strategies described here are straightforward, and when employed in the case of hemoglobin-oxygen binding reveal that the MWC model adequately accounts for heterotropic interactions in hemoglobin. These strategies can also be applied to other allosteric systems. Indeed, we here delineated a roadmap for successful data fitting to the MWC model so as to yield reliable mechanistic information necessary for understanding structure-function relations of an allosteric protein of interest.

## Supporting information

S1 FigThe concerted MWC allosteric model.(A) Schematic representation of the concerted MWC model applied to a tetrameric allosteric protein [11]. Square and round symbols respectively represent the tense (**T**) and relaxed (**R**) subunit conformations. *L*, *K*_T_ and *K*_R_ denote the **T** to **R** transition equilibrium constant in the absence of the substrate (S) and substrate affinity to the **T** and **R** conformations, respectively. The parameter *c* corresponds to the ratio of substrate affinity to the **R** and **T** conformations (= *K*_R_*/K*_T_). The fractional binding saturation of the MWC model (${\bar{Y}}_{MWC}$) considers all states depicted and may be given in the following traditional form:$\left({\bar{Y}}_{MWC}\right)=\left(\left(\left[\text{s}\right]/K_{\text{R}}\right){\left(1+\left(\left[\text{s}\right]/K_{\text{R}}\right)\right)}^{3}+L\left(\left[\text{s}\right]/K_{\text{T}}\right){\left(1+\left(\left[\text{s}\right]/K_{\text{T}}\right)\right)}^{3}\right)/\left({\left(1+\left(\left[\text{s}\right]/K_{\text{R}}\right)\right)}^{4}+L{\left(1+\left(\left[\text{s}\right]/K_{\text{T}}\right)\right)}^{4}\right)$. As indicated by Milo *et al*. [33], this equation can be re-parameterized based on the *Lc*^4^ and *LK*_R_^4^ compound parameters, respectively describing the transitions indicated by the red and green arrows (see SI Text in ref. [33]). (B) Theoretical dependence of the Hill coefficient at half-saturation (*n*_H_^MWC^) on the allosteric constant *L*, as determined according to Eq 4 in the Methods section [37]. The curve was plotted assuming a *c* value of 0.01. As can be seen, a bell-shaped dependence of *n*_H_ on *L* is obtained [12] with a maximal Hill value ($n_{H}^{max}$) of 2.76 obtained, given the indicated choice of *c*. The shallow region around the extremum point (where *Lc*^2^ = 1) [11] represents the ‘buffering of cooperativity’ region of hemoglobin [22], where changes in *L*, brought about by allosteric ligand binding affect only the affinity of oxygen binding to hemoglobin (*P*_50_), with no change in the slope of the binding isotherm, *i*.*e*., no change in cooperativity (*n*_H_).(TIF)

S2 Fig Steady-state analysis of the hemoglobin organophosphate physiological datasets using the traditional MWC model equation reveals compensatory, non-monotonic effects of *L* and *c* on effector concentration. The rigorous physiological datasets reported by Yonetani *et al*. [29] addressed the Bohr effect of hemoglobin in the presence of different organophosphate inhibitors. In this analysis, extremely accurate oxygenation curves were measured at different pH values and in the presence of different organophosphate effectors. Estimates for *L* and *c* for each curve, obtained using the traditional MWC equation, were reported in Fig 3 of reference [29]. (A-D) Dependence of the reported *L* (open circles) and *c* (solid circles) values of the Bohr effect saturation data of Yonetani *et al*. [29] in the absence (A) or presence of 2,3-BPG (B), IHP (Inositol hexaphosphate) (C) or BZF (bezafibrate) (D) organophosphate allosteric inhibitors. All datasets were measured in the presence of Cl^-^, as reported. In each case, no monotonic dependence of the reported *L* or *c* values on pH is observed. For each organophosphate dataset, as pH increases, *L* was first found to increase and then decrease. These changes are mirrored by opposite changes in *c*, primarily of *K*_T_. The MWC model [7,11] and its suggested modification, the global allostery model [29], do not provide a mechanistic explanation for this non-monotonic behavior. We thus suggest that the observed correlations between the *L* and *c* parameters in these datasets may reflect parameter adjustment, a result of data-fitting artifacts using the traditional MWC equation.
(TIF)

S3 Fig The three-equation system strategy is inadequate for assessing the effect(s) of physiological variations on allosteric protein function. (A-B) Correlation plot relating the observed (open circles) and calculated (filled circles) *n*_H_ values of the different oxygenation curves to either pH (A) or 2,3-BPG (B) effector concentration (S1 Table; see Methods and ref [34]). The pH and 2,3-BPG data reported here were respectively obtained at different [2,3-BPG] or [H^+^], as compared to the data presented in Fig 2 of the main text. (C-D) Dependence of the *L* (open circles) and *c* (solid circles) parameters of the physiological datasets on pH (C) and 2,3-BPG (D) effector concentration. A similar analysis for additional pH and 2,3-BPG physiological datasets, collected under different experimental conditions (see S1 Table), is presented in main text Fig 2.
(TIF)

S1 Table ‘TES strategy’-derived MWC parameters of human hemoglobin oxygen saturation curves of the physiological dataset^a^. ^*a*^The table reports the physiology-sound solution set from the TES strategy applied to different hemoglobin physiological datasets. ^*b*^The hemoglobin physiological datasets analyzed in the current study were complied from the meta-analysis described in ref. [33]. Values for *LK*_R_^4^ and *Lc*^4^ were reported therein, however, with no error bars indicated. ^*c*^Hill coefficients at half-saturation calculated based on the derived *K*_R_, *K*_T_, and *L* parameters (see text and Methods section). ^*d*^Hill coefficient at half-saturation derived upon fitting hemoglobin oxygen saturation data to the Hill equation, as reported in ref. [33]. ^*e*^data analyzed and presented in Fig 2. ^*c*^data analyzed and presented in S3 Fig.
(DOCX)

S2 Table ‘Global fitting’-derived MWC parameters of human hemoglobin physiological dataset saturation curves^*a*^. ^*a*^The MWC allosteric parameters for the hemoglobin pH, CO_2_ and 2,3-BPG physiological datasets were obtained using global fitting analysis, as described in the main text body and Methods section. ^*b*^The values for *L* are apparent, as they are determined in the presence of varying concentrations of the same effector. ^*c*^Hill coefficients at half-saturation calculated based on the *K*_R_, *K*_T_, and *L* MWC parameters (see text and Methods section). ^*d*^Hill coefficients at half-saturation derived upon fitting hemoglobin oxygen saturation data to the Hill equation.
(DOCX)
