# Supplementary figures and images for: Using the MWC model to describe heterotropic interactions in hemoglobin
Source: PLoS One. 2017 Aug 9;12(8):e0182871. doi: 10.1371/journal.pone.0182871 (PMC5549968; doi:10.1371/journal.pone.0182871)

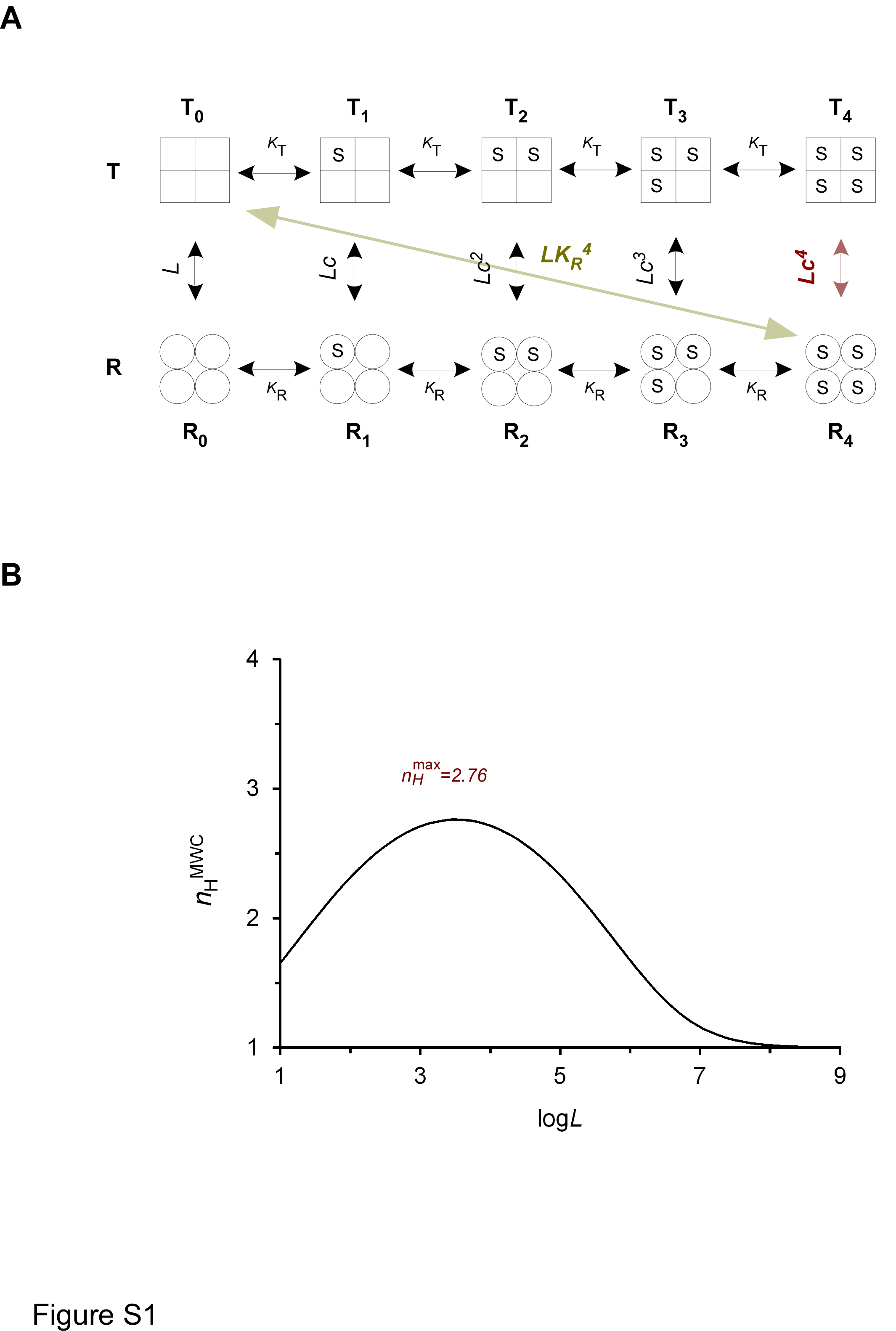

Supplement: S1 Fig — (A) Schematic representation of the concerted MWC model applied to a tetrameric allosteric protein [11]. Square and round symbols respectively represent the tense (T) and relaxed (R) subunit conformations. L, KT and KR denote the T to R transition equilibrium constant in the absence of the substrate (S) and substrate affinity to the T and R conformations, respectively. The parameter c corresponds to the ratio of substrate affinity to the R and T conformations (= KR/KT). The fractional binding saturation of the MWC model (Y¯MWC) considers all states depicted and may be given in the following traditional form:(Y¯MWC)=(([s]/KR)(1+([s]/KR))3+L([s]/KT)(1+([s]/KT))3)/((1+([s]/KR))4+L(1+([s]/KT))4). As indicated by Milo et al. [33], this equation can be re-parameterized based on the Lc4 and LKR4 compound parameters, respectively describing the transitions indicated by the red and green arrows (see SI Text in ref. [33]). (B) Theoretical dependence of the Hill coefficient at half-saturation (nHMWC) on the allosteric constant L, as determined according to Eq 4 in the Methods section [37]. The curve was plotted assuming a c value of 0.01. As can be seen, a bell-shaped dependence of nH on L is obtained [12] with a maximal Hill value (nHmax) of 2.76 obtained, given the indicated choice of c. The shallow region around the extremum point (where Lc2 = 1) [11] represents the ‘buffering of cooperativity’ region of hemoglobin [22], where changes in L, brought about by allosteric ligand binding affect only the affinity of oxygen binding to hemoglobin (P50), with no change in the slope of the binding isotherm, i.e., no change in cooperativity (nH). (TIF) [file pone.0182871.s001.tif]

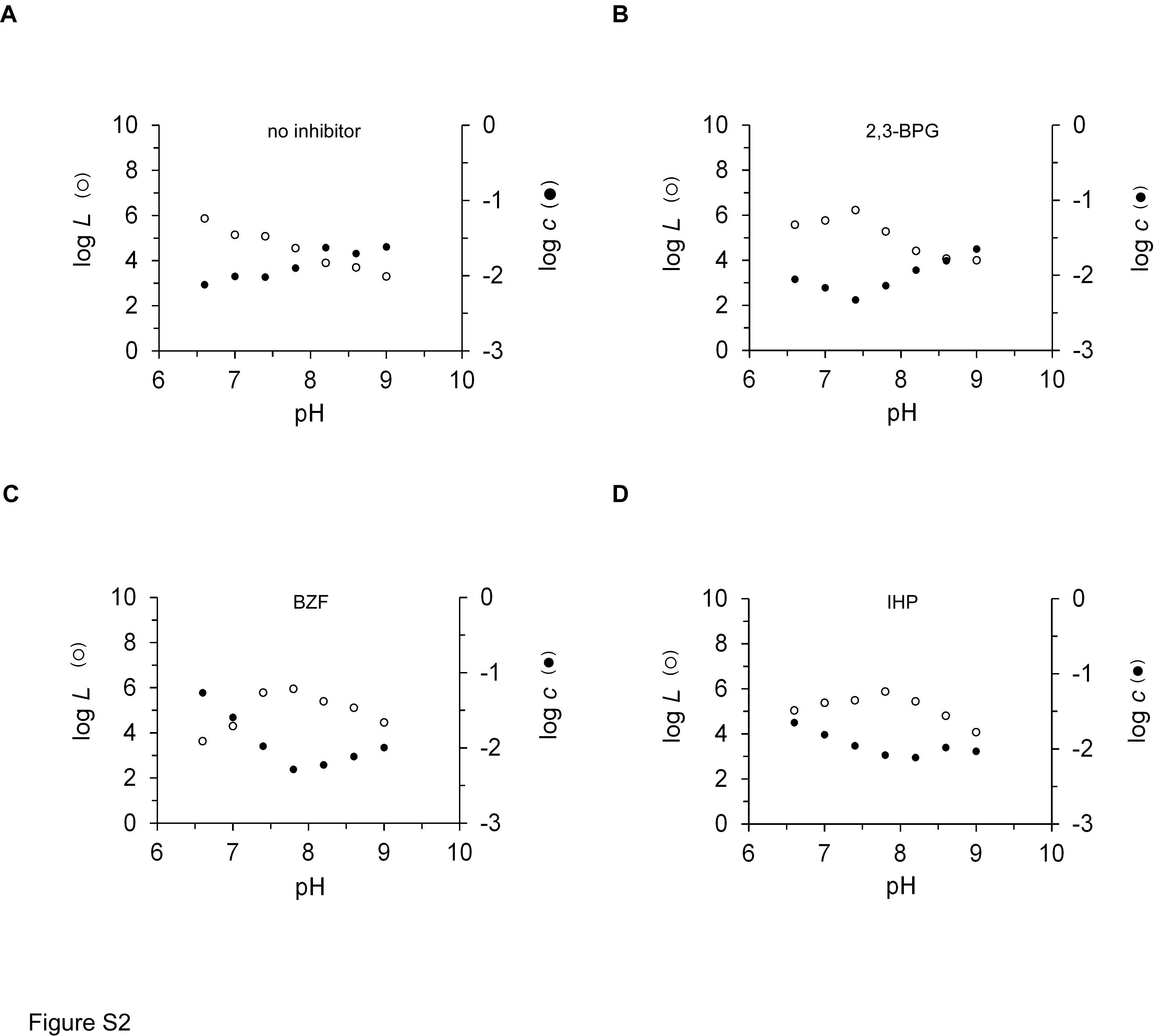

Supplement: S2 Fig — The rigorous physiological datasets reported by Yonetani et al. [29] addressed the Bohr effect of hemoglobin in the presence of different organophosphate inhibitors. In this analysis, extremely accurate oxygenation curves were measured at different pH values and in the presence of different organophosphate effectors. Estimates for L and c for each curve, obtained using the traditional MWC equation, were reported in Fig 3 of reference [29]. (A-D) Dependence of the reported L (open circles) and c (solid circles) values of the Bohr effect saturation data of Yonetani et al. [29] in the absence (A) or presence of 2,3-BPG (B), IHP (Inositol hexaphosphate) (C) or BZF (bezafibrate) (D) organophosphate allosteric inhibitors. All datasets were measured in the presence of Cl-, as reported. In each case, no monotonic dependence of the reported L or c values on pH is observed. For each organophosphate dataset, as pH increases, L was first found to increase and then decrease. These changes are mirrored by opposite changes in c, primarily of KT. The MWC model [7,11] and its suggested modification, the global allostery model [29], do not provide a mechanistic explanation for this non-monotonic behavior. We thus suggest that the observed correlations between the L and c parameters in these datasets may reflect parameter adjustment, a result of data-fitting artifacts using the traditional MWC equation. (TIF) [file pone.0182871.s002.tif]

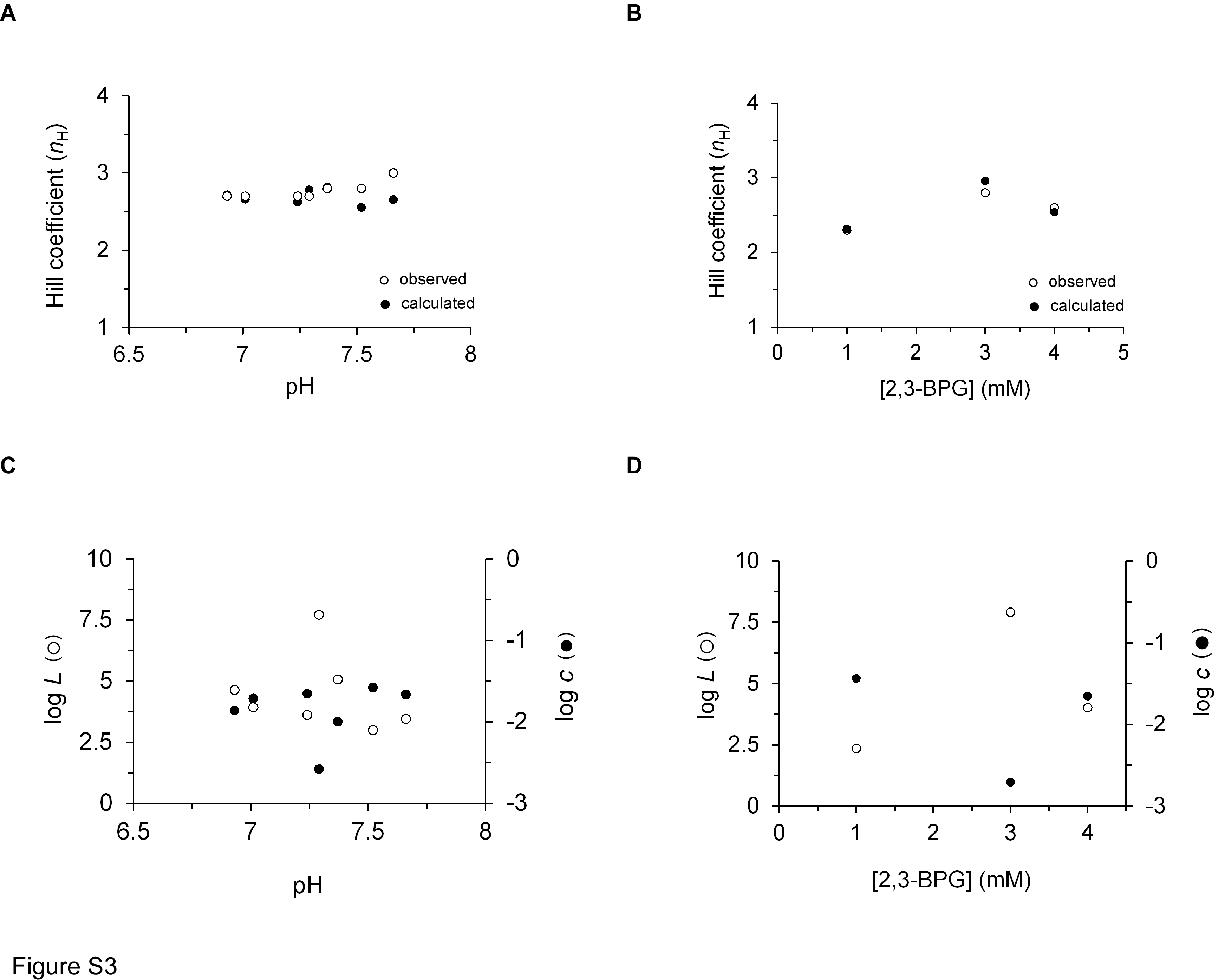

Supplement: S3 Fig — (A-B) Correlation plot relating the observed (open circles) and calculated (filled circles) nH values of the different oxygenation curves to either pH (A) or 2,3-BPG (B) effector concentration (S1 Table; see Methods and ref [34]). The pH and 2,3-BPG data reported here were respectively obtained at different [2,3-BPG] or [H+], as compared to the data presented in Fig 2 of the main text. (C-D) Dependence of the L (open circles) and c (solid circles) parameters of the physiological datasets on pH (C) and 2,3-BPG (D) effector concentration. A similar analysis for additional pH and 2,3-BPG physiological datasets, collected under different experimental conditions (see S1 Table), is presented in main text Fig 2. (TIF) [file pone.0182871.s003.tif]
