# Supplementary material for: Using the MWC model to describe heterotropic interactions in hemoglobin
Source: PLoS One. 2017 Aug 9;12(8):e0182871. doi: 10.1371/journal.pone.0182871 (PMC5549968; doi:10.1371/journal.pone.0182871)
Supplement: S1 Table — aThe table reports the physiology-sound solution set from the TES strategy applied to different hemoglobin physiological datasets. bThe hemoglobin physiological datasets analyzed in the current study were complied from the meta-analysis described in ref. [33]. Values for LKR4 and Lc4 were reported therein, however, with no error bars indicated. cHill coefficients at half-saturation calculated based on the derived KR, KT, and L parameters (see text and Methods section). dHill coefficient at half-saturation derived upon fitting hemoglobin oxygen saturation data to the Hill equation, as reported in ref. [33]. edata analyzed and presented in Fig 2. cdata analyzed and presented in S3 Fig. (DOCX) [file pone.0182871.s004.docx]

| **S1 Table. ‘TES strategy’-derived MWC parameters of human hemoglobin oxygen saturation curves of the physiological dataset^a^** | | | | | | | |
| --- | --- | --- | --- | --- | --- | --- | --- |
| *^b^***Physiology dataset** | ***Effector concentration*** | ***L x(*10*^5^)*** | ***K*_T_ (mmHg)** | ***K*_R_ (mmHg)** | ***c*** | ***n*_H_ (***^c^***calculated (MWC))** | ***n*_H_ (***^d^***observed)** |
|  |  |  |  |  |  |  |  |
|  |  |  |  |  |  |  |  |
| *^e^***pH** | **pH value** ([2,3-BPG] = 0.8 mM; P_CO2_ = 40 Torr) | | | | | | |
|  | 6.95 | 0.16 | 182.0 | 3.3 | 0.018 | 2.62 | 2.70 |
|  | 7.12 | 0.02 | 151.4 | 4.9 | 0.032 | 2.42 | 2.60 |
|  | 7.22 | 0.30 | 177.8 | 2.4 | 0.013 | 2.78 | 2.80 |
|  | 7.43 | 0.04 | 128.8 | 3.4 | 0.026 | 2.52 | 2.60 |
|  | 7.56 | 148.10 | 120.2 | 0.4 | 0.003 | 2.94 | 2.80 |
|  | 7.72 | 1.21 | 107.2 | 1.1 | 0.010 | 2.82 | 2.70 |
| *^f^***pH** | **pH value** ([2,3-BPG] = 0.8 mM; P_CO2_ = 70 Torr) | | | | | | |
|  | 6.93 | 0.40 | 204.2 | 4.0 | 0.013 | 2.72 | 2.70 |
|  | 7.01 | 0.08 | 177.8 | 3.9 | 0.019 | 2.66 | 2.70 |
|  | 7.24 | 0.04 | 134.9 | 0.4 | 0.022 | 2.63 | 2.70 |
|  | 7.29 | 527.8 | 151.4 | 1.5 | 0.003 | 2.78 | 2.70 |
|  | 7.37 | 1.18 | 182.0 | 4.8 | 0.010 | 2.82 | 2.80 |
|  | 7.52 | 0.01 | 151.4 | 3.3 | 0.026 | 2.55 | 2.80 |
|  | 7.66 | 0.03 | 151.4 | 3.3 | 0.021 | 2.65 | 3.00 |
| *^e^***2,3-BPG** | **[2,3-BPG] (mM)** (pH =7.1; P_CO2_ = 40 Torr) | | | | | | |
|  | 0.8 | 0.02 | 151.35 | 4.9 | 0.032 | 2.42 | 2.9 |
|  | 0.83 | 1.09 | 144.54 | 1.9 | 0.012 | 2.62 | 2.7 |
|  | 1 | 0.03 | 158.48 | 4.74 | 0.029 | 2.44 | 2.6 |
|  | 1.8 | 117.19 | 229.08 | 0.72 | 0.003 | 2.94 | 2.9 |
| *^f^***2,3-BPG** | **[2,3-BPG] (mM)** (pH= 7.3; P_CO2_ =40 Torr) | | | | | | |
|  | 0.40 | 0.01 | 177.8 | 6.5 | 0.036 | 2.32 | 2.30 |
|  | 1.00 | 0.10 | 134.9 | 3.0 | 0.022 | 2.54 | 2.60 |
|  | 1.80 | 817.04 | 190.5 | 0.37 | 0.002 | 2.96 | 2.80 |

*^a^*The table reports the physiology-sound solution set from the TES strategy applied to different hemoglobin physiological datasets

*^b^*The hemoglobin physiological datasets analyzed in the current study were complied from the meta-analysis described in ref. 33. Values for *LK*_R_^4^ and *Lc*^4^ were reported therein, however, with no error bars indicated.

*^c^*Hill coefficients at half-saturation calculated based on the derived *K*_R_, *K*_T_, and *L* parameters (see text and Methods section).

*^d^*Hill coefficient at half-saturation derived upon fitting hemoglobin oxygen saturation data to the Hill equation, as reported in ref. 33.

*^e^*data analyzed and presented in Fig 2

*^c^*data analyzed and presented in S3 Fig
