# Supplementary material for: Using the MWC model to describe heterotropic interactions in hemoglobin
Source: PLoS One. 2017 Aug 9;12(8):e0182871. doi: 10.1371/journal.pone.0182871 (PMC5549968; doi:10.1371/journal.pone.0182871)
Supplement: S2 Table — aThe MWC allosteric parameters for the hemoglobin pH, CO2 and 2,3-BPG physiological datasets were obtained using global fitting analysis, as described in the main text body and Methods section. bThe values for L are apparent, as they are determined in the presence of varying concentrations of the same effector. cHill coefficients at half-saturation calculated based on the KR, KT, and L MWC parameters (see text and Methods section). dHill coefficients at half-saturation derived upon fitting hemoglobin oxygen saturation data to the Hill equation. (DOCX) [file pone.0182871.s005.docx]

| **S2 Table. ‘Global fitting’-derived MWC parameters of human hemoglobin physiological dataset saturation curves*^a^*** | | | | | | |
| --- | --- | --- | --- | --- | --- | --- |
| **Physiological dataset** | **Allosteric effector concentration** | ***^b^L_app_*(10^5^)*** | ***c*** | ***n*_H_**  ***^c^*calculated (MWC)** | ***n*_H_**  ***^d^*Observed** | **Reference** |
| **pH** | **pH value** |  | | | | DiCera *et al*. (1988)  *J.Mol.Biol.* 200,  593-599 |
|  | 6.50 | 2.481±0.543 | 0.009±0.001 | 2.78±0.40 | 2.85±0.08 |  |
|  | 7.27 | 0.700±0.153 |  | 2.96±0.00 | 2.66±0.06 |  |
|  | 7.51 | 0.227±0.049 |  | 3.04±0.00 | 2.92±0.05 |  |
|  | 7.71 | 0.092±0.020 |  | 3.05±0.03 | 2.63±0.07 |  |
|  | 7.91 | 0.0485±0.010 |  | 3.02±0.00 | 2.72±0.06 |  |
|  | 8.50 | 0.009±0.002 |  | 2.85±0.29 | 2.62±0.05 |  |
|  | 9.10 | 0.005±0.001 |  | 2.74±0.06 | 2.24±0.06 |  |
| **pH** | **pH value** |  | | | | Imai, (1983)  *J.Mol.Biol*. 167,  741-749 |
|  | 6.70 | 14.981±3.617 | 0.007±0.001 | 2.63±0.00 | 2.77±0.08 |  |
|  | 7.20 | 1.706±0.415 |  | 3.02±0.12 | 2.64±0.08 |  |
|  | 7.65 | 0.148±0.036 |  | 3.14±0.02 | 2.58±0.06 |  |
|  | 8.00 | 0.030±0.007 |  | 3.05±0.19 | 2.59±0.06 |  |
|  | 8.20 | 0.007±0.002 |  | 2.85±0.09 | 2.56±0.05 |  |
|  | 8.80 | 0.004±0.0009 |  | 2.72±0.03 | 2.61±0.07 |  |
| **2,3-BPG** | **[2,3-BPG] (mM)** |  | | | | Benesch *et al*. (1971) *Nat.New Biol.* 8,  174 -176 |
|  | 0.00 | 0.030±0.022 | 0.014±0.001 | 2.87±0.02 | 2.32±0.12 |  |
|  | 0.10 | 0.033±0.024 |  | 2.87±0.12 | 2.67±0.16 |  |
|  | 0.25 | 0.057±0.041 |  | 2.88±0.03 | 2.75±0.12 |  |
|  | 0.40 | 0.091±0.065 |  | 2.87±0.04 | 2.72±0.13 |  |
|  | 0.60 | 0.129±0.093 |  | 2.85±0.00 | 2.94±0.18 |  |
|  | 1.00 | 0.159±0.114 |  | 2.84±0.46 | 2.75±0.16 |  |
|  | 2.50 | 0.295±0.212 |  | 2.78±0.17 | 2.83±0.19 |  |
| **CO_2_** | **pCO_2_ (Torr)** |  | | | | Doyle *et al.* (1987)  *J.Mol.Biol.* 196,  927-934 |
|  | 0.00 | 0.223±0.141 | 0.007±0.001 | 2.45±0.57 | 2.47±0.14 |  |
|  | 10.60 | 0.449±0.284 |  | 2.69±0.00 | 2.58±0.13 |  |
|  | 27.20 | 1.120±0.712 |  | 2.90±0.00 | 2.63±0.13 |  |
|  | 71.60 | 3.811±2.432 |  | 3.06±0.12 | 2.68±0.11 |  |
|  | 192.00 | 11.539±7.371 |  | 3.12±0.08 | 2.62±0.10 |  |
|  | 510.00 | 30.236±19.309 |  | 3.14±0.28 | 2.66±0.10 |  |

**S2 Table**

*^a^*The MWC allosteric parameters for the hemoglobin pH, CO_2_ and 2,3-BPG physiological datasets were obtained using global fitting analysis, as described in the main text body and Methods section.

*^b^*The values for *L* are apparent, as they are determined in the presence of varying concentrations of the same effector.

*^c^*Hill coefficients at half-saturation calculated based on the *K*_R_, *K*_T_, and *L* MWC parameters (see text and Methods section).

*^d^*Hill coefficients at half-saturation derived upon fitting hemoglobin oxygen saturation data to the Hill equation.
